# Isolated Fungal Balls in Urinary Bladder Presenting as Acute Retention of Urine

**DOI:** 10.1155/2020/4601474

**Published:** 2020-01-04

**Authors:** Tariq Hameed, Sudhir Kumar Jain, Faiz Manzar Ansari, Amrita Dua

**Affiliations:** Department of Surgery, Maulana Azad Medical College, Delhi, India

## Abstract

A 52-year-old male presented to surgery emergency with acute retention of urine. Patient was relieved in the emergency setting by catheterization and bladder irrigation. Urine was sterile; however, microscopy revealed field full of RBCs (>50/high-power field) and pus cells (>20/hpf). Cystoscopy revealed fungal balls in the urinary bladder which upon histopathological examination showed *Aspergillus* species. Patient was managed with systemic voriconazole and bladder wash with diluted povidone iodine. Predisposing factors diabetes mellitus and benign prostatic hyperplasia were medically managed, and patient recovered well. This case stresses the importance of considering isolated fungal urinary infections in predisposed individuals.

## 1. Introduction

Fungal ball in urinary bladder is an uncommon entity, the first case being reported way back in 1961 and around 20 cases have since then been reported in literature [1]. Tissue invasion of *Aspergillus* is rare and occurs most commonly in the setting of immunosuppression [2]. Isolated bladder infection with *Aspergillus* presenting as fungal balls is extremely rare [3]. Here, we present a case with urinary bladder fungal balls without disseminated disease or renal involvement who presented as progressive dysuria along with hematuria.

## 2. Case Presentation

A 52-year-old male presented to surgery emergency with acute retention of urine for 12 hours. He also complained of progressive dysuria and gross hematuria along with low-grade fever for the last 15 days. Patient had a history of lower urinary tract symptoms (LUTS) for the last 6 months. Patient was a shopkeeper by occupation with no known comorbidities or significant family history. On physical examination, patient was febrile with enlarged prostate on digital rectal examination and palpable bladder per abdomen. A Foley catheter was inserted, and bladder irrigation with diluted povidone iodine was started after obtaining urine samples.

**Figure 1:**
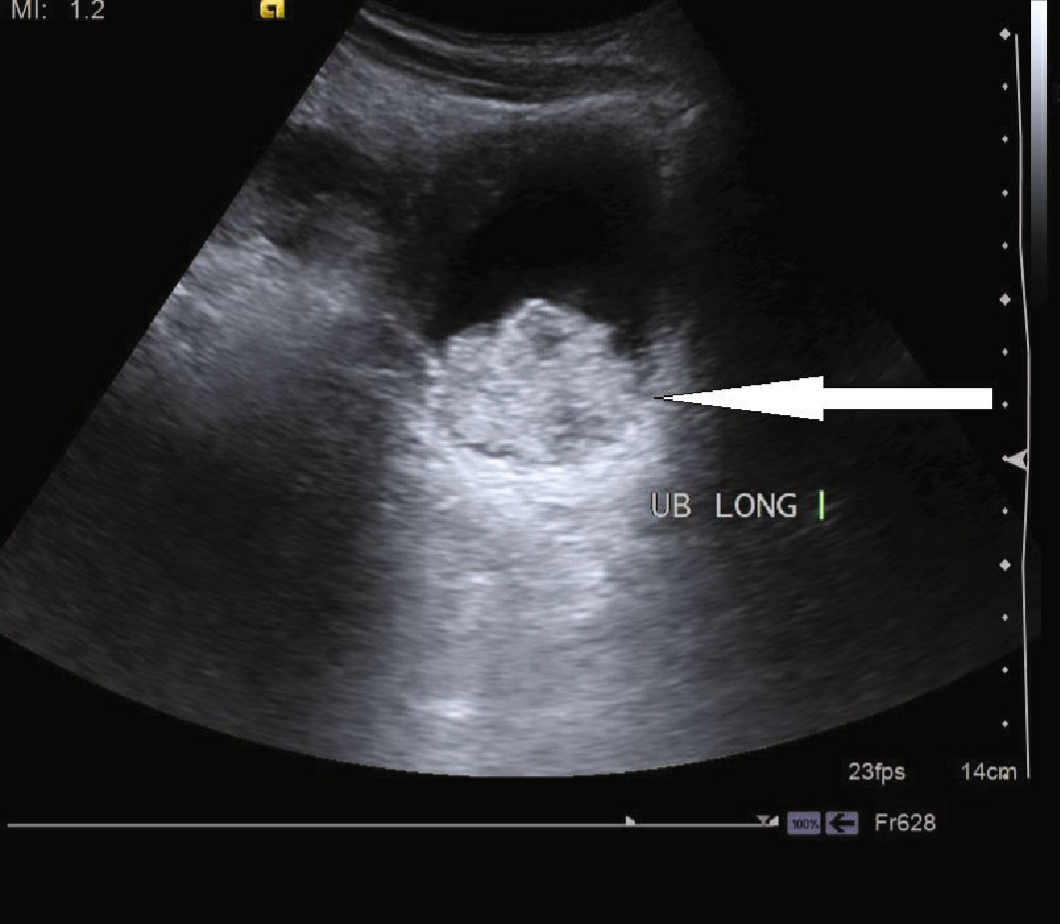
Ultrasonography showing hyperechogenic contents in the urinary bladder and thickened bladder wall.

Urine routine examination showed field full of RBCs (>50/high power field) and pus cells (>20/hpf). Urine culture was sterile. His WBC counts were 12,000/microlitre with absolute eosinophil count of 850. Blood sugar charting was deranged with fasting blood sugar 174 mg/dL and postprandial blood sugar 228 mg/dL. HbA1c was 11.2 g%. He was previously unaware of his diabetic status. USG abdomen showed hyperechogenic contents in the urinary bladder and thickened bladder wall ~7 mm (Figure 1); bilateral kidneys were normal without any abnormal echogenic focus or hydronephrosis.

**Figure 2:**
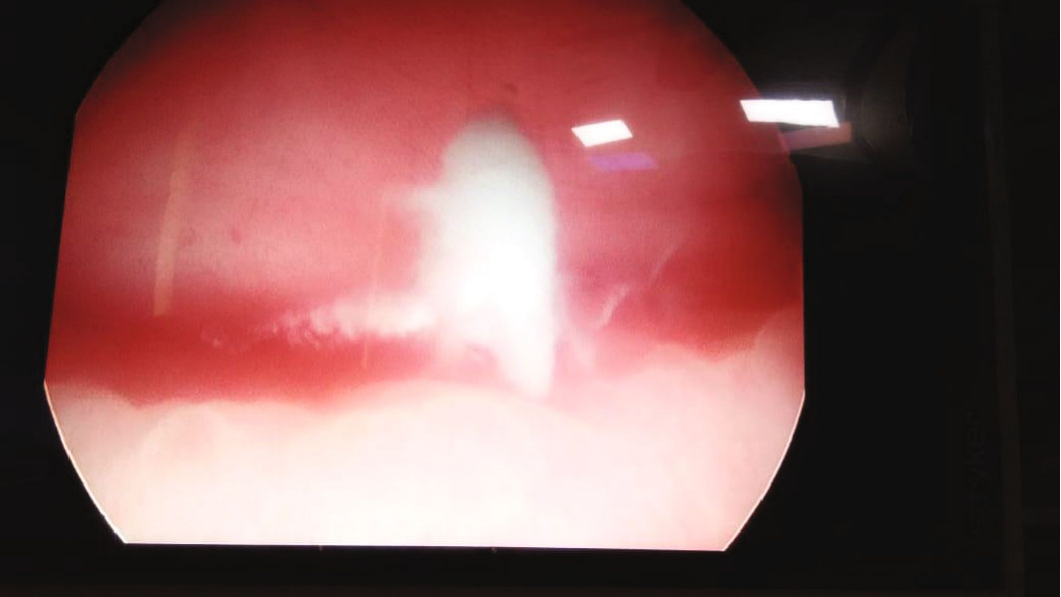
Cystoscopy showing fungal balls in the urinary bladder.

**Figure 3:**
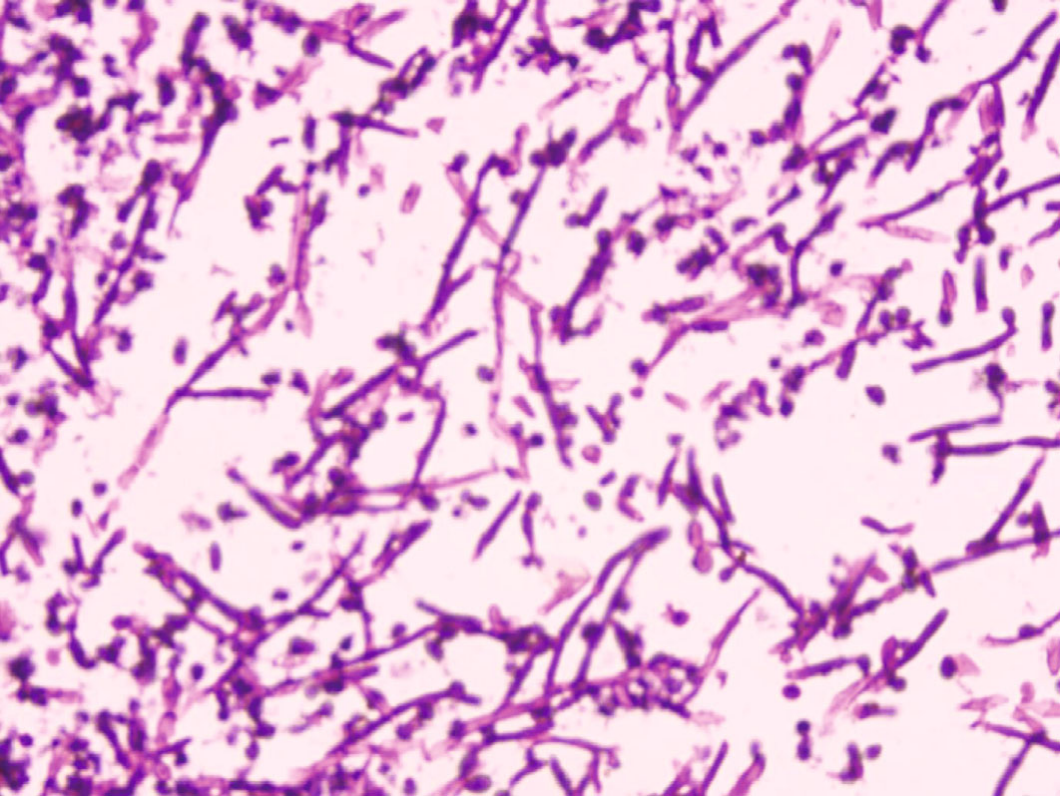
HPE showing septate fungal hyphae with acute angle branching.

To evaluate the cause of acute retention of urine, hematuria, and LUTS, patient was taken up for cystoscopy which showed membranous urethritis, median lobe prostatic enlargement, and white fluffy cotton-like balls in the bladder (Figure 2). Bladder wash fluid was sent for both bacterial and fungal culture, and fluffy balls were sent for histopathological examination (HPE). HPE showed fungal hyphae which were septate with acute angle branching and PAS stain positivity suggestive of *Aspergillus* (Figure 3). No growth was identified on Sabouraud's dextrose agar inoculation of bladder wash fluid. To screen the patient for immunodeficiency, an enzyme immune assay test for HIV 1 and 2 was done and found out to be negative. Additional tests for confirmation of *Aspergillus*-like galactomannan antigen and *β*-D-glucan were not performed in view of financial constraints for the patient. Patient was treated with systemic voriconazole and bladder irrigation with diluted povidone iodine. He was asymptomatic after 14 days of treatment. Check cystoscopy was performed after 4 weeks, and urinary bladder and urethra were normal without any evidence of fungal balls.

## 3. Discussion

*Aspergillus* are ubiquitous fungal molds found in organic matter. More than 100 species of *Aspergillus* have been identified while around 40 species are pathogenic to humans. Amongst them, *Aspergillus fumigatus* is the most commonly identified species. *Aspergillus* commonly affects the lungs, followed by the liver, spleen, heart, bones, central nervous system, ear, and paranasal sinuses [4, 5]. Fungal balls or bezoars are not common in the urinary bladder, and in majority of cases, *Candida albicans* has been found to be the causative agent [2, 6]. It is usually seen in immunocompromised states like hematological malignancies, in neutropenic patients following bone marrow or organ transplantation, or those receiving high dose of corticosteroids or cytotoxic drugs. Diabetes is also a risk factor for aspergillosis [7]. For urinary tract aspergillosis, additional risk factors are prolonged indwelling catheter, obstructive uropathy, or any surgical procedure. In the urinary tract, kidneys are most commonly involved, and isolated bladder involvement is very rare [3, 8, 9]. Diagnosis can be established through urine cytology and repeated fungal culture in case of resistant urinary tract infection (UTI) in predisposed individuals. In vitro fungal culture is positive in only around 50% of cases as fungus gets altered and is not viable, viability of fungal tissues in fungal balls is usually poor [10]. For invasive *Aspergillus* infection, galactomannan antigen and *β*-D-glucans are employed; they are quite sensitive and specific [11]. Managing urinary fungal infections is a tedious job. No definitive guidelines exist. Intravenous fluconazole/voriconazole, local irrigation with povidone iodine and amphotericin B, and cystoscopic removal of fungal balls and even operative methods are employed [12]. Under lying conditions like neurogenic bladder, diabetes mellitus, and prostatic hyperplasia must be taken care of.

## 4. Conclusion

Fungal infections of urinary tract even though rare should always be kept as differential diagnosis in patients of persistent UTI. In case of sterile pyuria with lower urinary tract symptoms, fungal cystitis should be considered as a possible diagnosis. Early diagnosis and complete treatment is required to control the dissemination of disease and to have a favourable prognosis. Fungal balls very rarely can also cause acute retention of urine in setting of severe lower urinary tract symptoms.

## References

[1] Chisholm E. R., Hutch J. A. (1961). Fungus ball (*Candida albicans*) formation in the bladder. *The Journal of Urology*.

[2] Irby P. B., Stoller M. L., McAninch J. W. (1990). Fungal bezoars of the upper urinary tract. *The Journal of Urology*.

[3] Sakamoto S., Ogata J., Sakazaki Y., Ikegami K. (1978). Fungus ball formation of aspergillus in the bladder. An unusual case report. *European Urology*.

[4] Raja N. S., Singh N. N. (2006). Disseminated invasive aspergillosis in an apparently immunocompetent host. *Journal of Microbiology, Immunology, and Infection*.

[5] Soubani A. O., Chandrasekar P. H. (2002). The clinical spectrum of pulmonary aspergillosis. *Chest*.

[6] Kauffman C. A. (2005). Candiduria. *Clinical Infectious Diseases*.

[7] Merseburger A. S., Oelke M., Hartmann J., Stenzl A., Kuczyk M. A. (2004). Intracranial aspergillosis in a non-immunocompromised patient treated for muscle-invasive bladder cancer. *International Journal of Urology*.

[8] Dervisoglu E., Dikmen E., Filinte D., Yilmaz A. (2008). Isolated bladder aspergillosis as the primary presentation of non-oliguric acute renal failure. *Scandinavian Journal of Urology and Nephrology*.

[9] Siddappa S., Mythri K., Kowsalya R., Shivalingaiah M. (2012). An unusual case of non-disseminated bladder aspergillosis in a setting of transitional cell carcinoma. *Indian Journal of Medical Microbiology*.

[10] Willinger B., Obradovic A., Selitsch B. (2003). Detection and identification of fungi from fungus balls of the maxillary sinus by molecular techniques. *Journal of Clinical Microbiology*.

[11] Desoubeaux G., Bailly E., Chandenier J. (2014). Diagnostic de l'aspergillose pulmonaire invasive : actualites et recommandations. *Médecine et Maladies Infectieuses*.

[12] Lechmiannandan S., Goh E. H., Teoh B. W., Git K. A. (2012). A rare case of fungus balls of the urinary bladder due to Candida Tropicalis. *UroToday International Journal*.

